# Time series traffic collision analysis of London hotspots: Patterns, predictions and prevention strategies

**DOI:** 10.1016/j.heliyon.2024.e25710

**Published:** 2024-02-10

**Authors:** Mohammad Balawi, Goktug Tenekeci

**Affiliations:** a Cyprus International University, North Nicosia, North Cyprus; b Jacobs; and Cyprus International University, North Nicosia, North Cyprus

**Keywords:** Traffic collisions, Traffic accidents, Time series analysis, Accident frequency forecasting, Safety measures, ARIMA model, SARIMAX model

## Abstract

Despite recent measures on accident prevention, road collisions, mainly on London's “A" roads, persist as accident sources, endangering vulnerable users in particular. Analysing evidence from London's A-Roads unveils issues concerns and trends. This study utilises extensive data to target factors magnifying accidents: speed, traffic, vulnerable interactions. Stats 19 and transport data including volumes, types, speeds, and congestion parameters are all analysed alongside the collision data. The descriptive statistics have been employed to understand nature of data in the first instance. This has supported the process to cleanse the data outliers or periods where were subjected to incidents and interventions. Predictive model development is conducted to analyse and forecast accident frequency using ARIMA and SARIMAX models forecasted accident rates and interventions. ARIMA yielded higher accuracy. Method of analysis resulted in a statistically reliable formulation of the main factors, enabling use of this method for similar cities across the world. Formulated analysis revealed key contributors as population density, weather, and time of the day. The analysis of data supported identification of strategies emerging as infrastructure improvements, traffic control measures and severity and vulnerable users affected in particular. The analysis reveals distinct exhibits of causation, leading to focused recommendations on infrastructure enhancements, traffic control measures, and the impact on severity and vulnerable users, deviating from prior research findings. Insights aid safer London roads, have global predictive and mitigation value.

## 1 Introduction

A road traffic accident involves a road vehicle in an incident leading to injury or death on a public road [1]. Road accident causation is complex, involving driver behaviour, infrastructure, vehicle traits, economy, and environment. These factors interact to affect accident occurrence and severity. Yet, transport's importance has increased over five decades and continues to increase for access, jobs, education, services, and goods. Despite improvements, road accidents remain a leading global cause of fatalities.

Globally, traffic collisions raise significant safety and economic concerns. The World Health Organization notes 1.35 million deaths and 50 million injuries annually from road accidents [2]. Vehicle numbers align with population growth. By 2030, road traffic accidents are projected by WHO to become the 7th leading cause of fatalities [3,4]. Vulnerable road users, like cyclists, motorcyclists, and pedestrians, comprise 54% of road fatalities. Various factors, including human error, vehicle defects, and environmental conditions, have been identified as major contributors to road traffic accidents [5]. Addressing these is vital for global road safety. WHO's 2015 data highlights disparities: low-income countries saw 24.1 fatalities per 100,000, middle-income 18.4, high-income 9.0. Lower-income countries contribute over twice the fatalities of higher-income ones [3].

The transport sector's decarbonisation is crucial for addressing energy security, environmental concerns, and climate change. Karkatsoulis et al. (2017) presented a model to simulate deep Greenhouse Gas (GHG) emission reductions in transport, identifying both positive industrial impacts and the economic implications of freight and passenger transport costs [6]. Focusing on London, Zanni and Bristow (2010) analysed road freight CO₂ emissions trends and underscored the need for profound behavioural measures to meet the city's emission targets [7]. Together, these studies highlight the multifaceted challenges and opportunities in decarbonizing the transport sector across different regions alongside the accident problems.

The severity of accidents can increase due to a combination of factors. Some common causes that contribute to the increased severity of accidents include [8].

1. **Speed:** as the force of the collision is greater, resulting in increased risk of injury or fatality.
2. **Vehicle Size and Type:** tend to cause more severe accidents when involved in collisions with smaller vehicles.
3. **Behaviour:** such as speeding, distracted driving, impaired driving (due to alcohol or drugs), or failure to follow traffic rules.
4. **Road Conditions:** inadequate signage, lack of proper lighting, uneven surfaces, or insufficient road maintenance contributes to accidents and increase in severity.
5. **Lack of Safety Features:** lack of advanced driver assistance systems (ADAS), may result in more severe injuries during accidents.
6. **Vulnerable Road Users:** pedestrians, cyclists, or motorcyclists, often suffer more serious injuries due to their lack of protection compared to occupants of enclosed vehicles.
7. **Emergency Response Time:** Delays in emergency response and medical assistance can impact on the severity of accidents, as prompt medical attention can significantly improve outcomes for injured individuals.

The severity of accidents can be influenced by a combination of these factors, and understanding causes on each road segment would help addressing them or reducing severity through improved road infrastructure, driver education, enforcement of traffic laws, and vehicle safety measures.

The avoidance of road traffic accidents has become a transportation management priority. Using various data mining techniques, initial and secondary collisions can be determined and studied to aid transport authorities and contribute to reducing the high number of roadway injuries and fatalities [ [9,10,11,12], and [13]].

Road safety is a paramount concern for societies worldwide, with a pressing need to understand and mitigate the factors contributing to accidents. In this study, we delve into the intricate dynamics of road safety, focusing on a specific geographic area encompassing four major roads: A1, A3, A4, and A6. Our investigation spans the critical four-year period from 2016 to 2019, allowing us to analyse a comprehensive dataset and gain insights into the unique challenges and patterns associated with these roadways. Through rigorous statistical analysis and modelling, we aim to unravel the key determinants of accidents and provide a predictive framework that can enhance road safety measures.

London's road traffic accidents continue to be a significant problem, with a high proportion of accidents occurring on A-roads, primary roads, and urban major roads. According to recent statistics, approximately 60% of all road traffic accidents in London occur on these roads, with a significant number of accidents resulting in injuries or fatalities. Addressing this issue is crucial to ensure the safety and well-being of all road users and to create a more sustainable and efficient transportation system in London.

This study aims to comprehensively analyse the factors influencing road accidents on London's A-Roads, focusing on elucidating the primary causes and developing predictive models for informed interventions. The objectives encompass identifying influential variables such as weather conditions, time of day, vehicle type, and driver behaviour. The rationale for this research lies in the need to enhance road safety measures where despite recent significant safety improvements overall, heavily trafficked A-roads which exhibit composition of active and other modes, retains the significantly higher accidents. An area where no publications are made to date.

The approach harnesses advanced statistical models—particularly ARIMA and SARIMAX—augmented by comprehensive data sourced from the Department for Transport's Road Safety Data. By unveiling critical insights, this study presents a novel perspective on accident prediction, emphasizing infrastructure improvements, traffic control measures, severity impact, and vulnerability to specific road users. The strengths of our study stem from its rigorous methodology, encompassing sophisticated statistical techniques and the utilization of comprehensive, publicly available datasets. The interpretation of our results is underpinned by the rigorous analysis conducted, supporting the conclusions drawn regarding the identified strategies and their implications for road safety. Furthermore, the reproducibility of our approach serves as a template for similar studies, providing a robust framework for analysing and predicting accidents on various road networks.

This study's primary objectives are to.

1. Identify the characteristics of road hazards on Primary (A-Roads) in London,
2. Formulate the causation factors and main contributors to accident occurrence, through constructing a time series models to predict the rate of road traffic accidents in London where an in-depth analysis of variables are then related to the severity of accident rates
3. Investigate the impact of safety measures of roads in reducing the rate of traffic accidents,
4. In addition, the research investigates the effectiveness of the main road safety measures implemented during the study period in reducing the number of accidents.

The hypothesis established are:

This study aims to investigate and validate critical hypotheses concerning the persistent challenges surrounding traffic accidents prevalent on heavily trafficked A-roads. Firstly, the research focuses on the enduring significance of specific environmental conditions, particularly the impact of wet and dark (absence of natural light) conditions, hypothesized to substantially contribute to the causation of accidents. These factors, previously reported in literature, are examined within the context of A-roads, emphasizing their continued relevance and influence on accident rates. Secondly, despite sustained enforcement efforts and comprehensive safety campaigns, the study aims to explore the sustained influence of high-speed driving behaviors on the occurrence of accidents within the specified area. This hypothesis seeks to validate the notion that despite regulatory measures, high-speed driving practices continue to be a substantial factor contributing to accidents on these roads. Lastly, the research posits that the absence of segregated facilities or the composition of various transportation modes in densely trafficked conditions significantly impacts accident causation. This hypothesis aims to explore if the infrastructure layout and mixed transport modes prevalent on A-roads might contribute to accident rates, addressing an area that might have been overlooked in prior studies within similar settings.

With the development of effective forecasting models and descriptive statistics of traffic safety data, it is anticipated that the following questions will be answered.

1. Which models to be developed provides a more reliable forecasting method for predicting the rate of accidents?
2. What are the most important variables that explain the number of accidents?
3. How effective are London's road safety measures at decreasing the traffic accidents rate?

The outcome of the analysis is aimed at supporting transport professionals involved in road safety in predicting the future pattern of road traffic accidents, allowing them to take appropriate action to minimise and avoid death toll by appropriate measures implementation.

### 1.1 Limitations

1. **Data Continuity:** Whilst ideally a five-year is a norm for accident analysis the research data spans from 2016 to 2019 due to the impact of the COVID-19 pandemic, preventing the collection of data for more recent years. This limitation could be acknowledged by emphasizing the constraints posed by the pandemic on data collection and the subsequent analysis. However, in mitigation, data is utilized for entire length of all A-Road corridors in London
2. **Missing Features:** The dataset obtained from the UK Department of Transport lacks certain crucial features like traffic flow, which could have enhanced the comprehensiveness of the analysis. Mitigating this limitation involved analysis of traffic flows published by Department of Transport for the entire corridors.
3. **Data Scope:** The decision to focus the analysis solely on London due to the large dataset representing the entire UK could be justified based on feasibility and manageability. This choice could be presented as a practical solution to handle voluminous data and ensure a more detailed, localized analysis.

## 2 Background

Road traffic accidents continue to pose significant challenges worldwide, necessitating advanced predictive models and methodologies to enhance road safety. This part contains a review of the literature from extensive sources relevant to traffic and road safety studies that focus on the factors that affect safety and accident analysis and prediction models with a comprehensive review of existing research illuminates various methodologies and limitations in this domain.

Dong et al. presented an innovative deep learning model for traffic crash prediction, highlighting its potential in identifying relationships between variables and crash occurrences. However, limitations were noted regarding unavailable factors leading to biased estimations and inaccurate predictions [14]. Therefore, this research essentially incorporated additional factors into models for reduced bias and improved predictions. The gap was addressed by incorporating a more extensive dataset and employing advanced machine learning techniques that accounted for missing or noisy data, refining predictions, and reducing bias.

Ihueze and Onwurah [15] developed predictive models using AutoRegressive Integrated Moving Average (ARIMA) and Seasonal AutoRegressive Integrated Moving Average with eXogenous variables (SARIMAX) models to forecast crash frequency in Nigeria, highlighting eleven significant factors affecting crash occurrence and crash frequency, emphasizing the significance of human, vehicle, and environmental factors. Nevertheless, their study was limited to a specific region, hindering generalizability. Also, the impact of certain factors like mobile phone usage and demographics was overlooked and extending the research to encompass other regions, considering additional crash-contributing factors is limited. These limitations were addressed by utilizing a broader dataset covering multiple regions in London and incorporating various factors to enhance the model's robustness.

La Torre et al. [16] focused on developing an Accident Prediction Model (APM) specifically for Italian freeways. Developed accident prediction models based on Safety Performance Functions and Crash Modification Factors, effectively identifying high-risk areas for safety interventions. Their study, though valuable, was constrained to rural freeway segments in Italy and considered only a limited number of factors, potentially impacting generalizability and comprehensiveness. Investigating the applicability to diverse road types and countries, incorporating more factors into the models. These gaps were addressed in this research by exploring diverse road types, countries, and incorporating a broader range of factors for more comprehensive accident prediction models.

Labib et al. [17] conducted a comprehensive analysis of road accidents in Bangladesh using machine learning techniques. While achieving an 80% accuracy, the study's limitations included reliance on historical data and a focus on a limited set of factors, potentially limiting the model's predictive capacity and the gap was addressed in identifying additional factors contributing to accidents, exploring non-machine learning approaches for analysis. Further expansion was undertaken in this research by incorporating newer datasets, identifying additional accident-contributing factors, and exploring diverse analytical approaches beyond machine learning.

Zhao et al. [18] introduced a traffic accident prediction model based on Convolutional Neural Networks (CNNs). However, the study's limitations included dataset representativeness issues and reliance on accurate input traffic data. Therefore, the research is in need for more comprehensive datasets, real-world testing of the algorithm, and addressing ethical considerations. This research aimed to gather diverse datasets, evaluate the real-world feasibility of the algorithm, and contemplate ethical implications for the implementation of such predictive models.

Bai et al. [19] proposed a method for identifying ARX models (Autoregressive with Extra Input) with multi-Gaussian noises. However, the assumed noise models might not always represent real-world scenarios, limiting the algorithm's applicability and exploring the algorithm's adaptability in diverse real-world scenarios. Exploration of more diverse noise models and scenarios was aimed by this research to enhance the adaptability and applicability of the algorithm.

Santos et al. [20] explored machine learning approaches for traffic accident analysis. However, the study highlighted limitations regarding data quality and potential biases in machine learning algorithms and development of more accurate machine learning algorithms, standardization of data collection methods. This research aimed to refine machine learning algorithms, improve data quality, and standardize data collection to ensure more reliable predictions.

Zhang et al. [21] presented a road traffic accident prediction model based on LSTM-GBRT, noting limitations related to data collection and forecasting ability and improving relevant feature collection and enhancing forecasting ability. Avenues were proposed in this research to gather comprehensive features and enhance the predictive capacity for traffic accident forecasting.

Jamal et al. [22] conducted a comparative study on injury severity prediction of traffic crashes. The study lacked considerations for temporal and spatial variations in crashes. The gap was addressed in addressing data imbalance issues and exploring various machine learning techniques.

The gaps identified in Jamal et al.'s study might have been addressed in this research by potentially addressing data imbalance, exploring diverse machine learning techniques, and considering temporal and spatial variations in the number of vehicles in accident prediction models for traffic crashes.

Rahim & Hassan [23] introduced a deep learning-based traffic crash severity prediction framework, acknowledging potential biases and limitations in data representativeness. The gap was identified in need for more comprehensive crash data and exploring alternative image transformation techniques. Diverse image transformation methods were explored in this research to improve predictive accuracy, aiming to enhance data representativeness using ARCMAP.

Wang et al. [24] provided a comprehensive review of mid-term load forecasting, but the review lacked discussions on the limitations of reviewed methods and ethical implications and exploration of long-term load forecasting and addressing ethical considerations. Long-term load forecasting methods were investigated in this research, aiming to assess ethical implications of predictive methodologies.

Overall, these studies employed diverse methodologies for traffic accident prediction, offering insights to policymakers and practitioners for better road safety strategies. Below table introduces a summary of methods with limitations and advantages.

Table 1 provides a summary of various models employed by different researchers for predicting and analysing traffic accidents. The table provides a comprehensive overview of the strengths and weaknesses of different approaches to traffic accident prediction, offering insights into their applicability, limitations, and reported performance. A range of techniques employed, from traditional time series models like ARIMA to advanced deep learning models, to address various aspects of road safety and accident prediction.

**Table 1:** A summary table of the models mentioned.

Researcher & Model	Approach/Methodology	Advantages	Limitations	Reported Significant Findings & Confidence Level
Dong et al., 2018	Deep Learning-based Prediction Model	Improved road safety, accurate prediction	Requires substantial data and computational resources	High confidence in predicting accident trends, moderate confidence in predicting specific accident types
Ihueze & Onwurah, 2018	ARIMA and ARIMAX for Crash Frequency Prediction	Accurate predictive models, insight into contributing factors	Limited to crash frequency prediction	High confidence in identifying seasonal patterns, moderate confidence in identifying other contributing factors
La Torre et al., 2019	Juris Directional SPF-based Accident Prediction Models	Solid and reliable tool for predicting accidents, prioritizing safety interventions	Specific to Italian freeways, may not generalize well	High confidence in predicting accident rates on Italian freeways, low confidence in applicability to other contexts
Labib et al., 2019	Machine Learning for Traffic Accident Analysis	Identifies significant factors, provides suggestions for road safety interventions	Focuses on analyzing accident severity, may not cover prediction	High confidence in identifying contributing factors to severe accidents, moderate confidence in recommending interventions
Zhao et al., 2019	CNN-based Traffic Accident Prediction Model in VANET	Efficient feature extraction, accurate prediction	Dependent on data availability in VANET, may require significant data preprocessing	High confidence in predicting accident patterns in VANET, moderate confidence due to data limitations
Bai et al., 2019	Enriched SARIMAX Predictions with Exogenous Variables	Improved prediction accuracy	Dependent on availability and accuracy of exogenous variables	High confidence in integrating exogenous factors to enhance prediction accuracy, low confidence in variable selection
Zhang et al., 2020	LSTM-GBRT for Road Traffic Safety Prediction	Good predictive ability, robustness	Specific to road safety level prediction	High confidence in predicting general road safety trends, moderate confidence in specific event prediction
Santos et al., 2021	Machine Learning for Accident Hotspot Prediction	Effective in predicting accident hotspots, informs road safety measures	Focuses on predicting accident hotspots, may not cover overall accident prediction	High confidence in hotspot prediction accuracy, moderate confidence in overall accident prediction
Jamal et al., 2021	Ensemble Machine Learning for Injury Severity Prediction	Improved accuracy in predicting injury severity, early warning system potential	Focuses on injury severity prediction, may not cover overall accident prediction	High confidence in predicting injury severity levels, low confidence in overall accident prediction
Rahim & Hassan, 2021	Deep Learning for Work Zone Crash Severity Prediction	Improved prediction of rare classes, accuracy enhancement	Specific to work zone crash severity prediction	High confidence in predicting rare crash types in work zones, moderate confidence in overall severity prediction
Wang et al., 2022	Enhanced SARIMAX with Weather Variables	Improved prediction accuracy with weather integration	Dependent on availability and accuracy of weather data, may increase model complexity	High confidence in weather's impact on accident rates, moderate confidence in predicting specific weather-related incidents

Differing from prevailing methodologies, this research distinguishes itself by leveraging an extensive and comprehensive dataset spanning over a period of 4 years. The dataset encompasses detailed records across a network of 4 corridors within the specified area, providing a more in-depth view and a comprehensive spatial representation of traffic accidents occurring in these heavily trafficked corridors. This inclusive approach allows for a more nuanced analysis and a deeper understanding of accident patterns and contributing factors across a diverse range of geographical locations and temporal periods. Specifically focusing on the geographic expanse of London's primary road network (A-Roads), a scope unexplored in prior reported papers. This expansion substantially elevates the reliability and representativeness of our study findings. Notably, the adopted approach for this study approach transcends the conventional reliance on machine learning or artificial intelligence methodologies, pivoting instead towards empirical and data-driven analysis. Consequently, the derived outcomes from this study yield novel insights, particularly emphasizing a clearer understanding of the pronounced effects of adverse weather conditions (wet and dark, i.e., absence of natural light), high-speed driving, and the composition of traffic in heavily trafficked areas. These findings directly correlate with the hypothesized impact of these factors on the occurrence of traffic accidents, reinforcing their significance and shedding new light on their multifaceted influences. These findings underscore the transformative impact of cutting-edge safety interventions, highlighting a paradigm shift in safety enhancement strategies.

The long-term effects of published research have enabled adopting a lessons-learned approach for data gathering, sifting for accuracy and analysis method, leading to development of a prediction model. It starts by describing what road traffic safety is and some basic notions concerning the causes of road accidents. It is expected to familiarize the reader with the fundamental assumptions about problem solving that went into the research design and interpretation.

Additionally, researchers have employed statistical analysis in establishing reliability of data. These methods in brief have been.

• The Augmented Dickey Fuller (ADF) test examines time series for unit roots, assessing stationarity—a vital assumption for many time series models. It contrasts the null hypothesis of a unit root (non-stationarity) against the alternative of stationarity. Dickey and Fuller introduced this test [25,26].
• The Autocorrelation Function (ACF) and Partial Autocorrelation Function (PACF) are essential tools in time series analysis. ACF gauges correlation between a series and its lags, signaling past values' influence. PACF accounts for intermediate lags. Both assist in determining autoregressive and moving average terms [27,28].

These techniques, namely ADF, ACF, and PACF prove pivotal in analyzing stationarity and autocorrelation, shaping reliable time series models. By understanding data's statistical attributes, experts can aptly select models, estimate parameters, and forecast in diverse domains like finance, economics, and engineering.

This study references a variety of data types related to the exposure, risk, and damage caused by traffic accidents on roads. These statistics encompass historical estimated road traffic casualties by type of road user, demographic information, the number of registered vehicles, financial status, infrastructure, and meteorological information. The primary source of this data is the Road Safety Statistics (STATS19) provided by Department of Traffic in the UK.

The STATS19 is the official road safety statistics data set in the UK, starting from 1979. It records personal injury accidents on public roads that are reported to the police and then documented using the STATS19 accident reporting form. The data within STATS19 is coded, and to understand these codes, lookup tables provided in the supporting papers section were utilized. It's crucial to note that the data also includes adjustments for casualty and collision severity, accounting for changes in how the police report severity [29].

Based on review of data, limitations of statistical tools and fit-for purpose of this study, various tools (Table 2): such as Microsoft Excel 2016 for data presentation and graphing, SPSS for statistical analysis, and GIS for location-based data are deemed necessary. GIS records Earth's spatial-temporal references using coordinates. Python, a versatile language, supports diverse programming paradigms, known for readability and a rich library. ArcGIS aids mapping, visualization, and analysis.

**Table 2:** Summary table of the software tools used in the study and their respective uses.

Software	Use
Microsoft Excel 2016	Microsoft Excel 2016 is a spreadsheet application used for data organization, basic statistical computations, graphing, and scripting. It's a popular tool for data manipulation and visualization.
SPSS	SPSS (Statistical Package for the Social Sciences) is widely used for statistical analysis. Popular in fields like social science, healthcare, and education, it offers tools for data management, descriptive and inferential statistics, and data documentation.
ArcMap (Version 10.8)	ArcMap is part of the ArcGIS software suite for geographic information system (GIS) analysis. It integrates spatial and temporal data, enabling the connection of location-based data with other datasets.
Python	Python is a versatile programming language employed for diverse data analysis purposes. Its extensive libraries and frameworks enable tasks like data manipulation, statistics, machine learning, and visualization.

In the studies mentioned previously, a variety of software tools were harnessed to conduct comprehensive analyses and derive meaningful insights. These tools played pivotal roles in data processing, statistical analysis, and visualization. Below is a summary of the software tools employed in those studies and their respective applications in Table 2.

After a comprehensive review of various statistical and analytical methods discussed above, it becomes evident that both the ARIMA and SARIMAX models are well-suited to the objectives of this study. The ARIMA model's strength lies in its ability to capture time-dependent patterns and trends, which is particularly relevant when analysing time series data such as traffic accidents. Moreover, the model provides a clear framework for addressing seasonality and autocorrelation, factors that can significantly impact accident prediction accuracy. On the other hand, the SARIMAX model introduces the capability to incorporate exogenous variables, which can further enhance its predictive power by considering external factors that influence accidents. Both models strike a balance between complexity and predictive accuracy, and their ability to accommodate seasonality and exogenous variables makes them ideal choices for this study.

## 3 Methodology

The approach followed in this paper is based on following principles.

• Solid research relies on sound data for analysis. We acquired UK Traffic data (2016–2019) on accidents, meticulously cleaned and refined over three years to ensure quality and size. This prepares us to study factors influencing London's traffic accidents. Key variables are identified for predictive accident models.
• Our effective traffic accident analysis involves data cleaning, explanatory time series modelling, and influential feature selection. ARIMA and SARIMAX models capture patterns. Historical accident data, including weather, informs predictions. Rigorous model evaluation ensures reliability, revealing insights for road safety enhancements.

### 3.1 Data

For our research, we will be focusing on specific corridors within London, namely A-roads, primary roads, and urban major roads. These corridors have been chosen due to their significant contribution to the overall traffic volume and the higher proportions of accidents. This study employs a period of four years of data, from 2016 to 2019, allowing for a comprehensive analysis of trends and patterns over time.

**Fig. 1:**
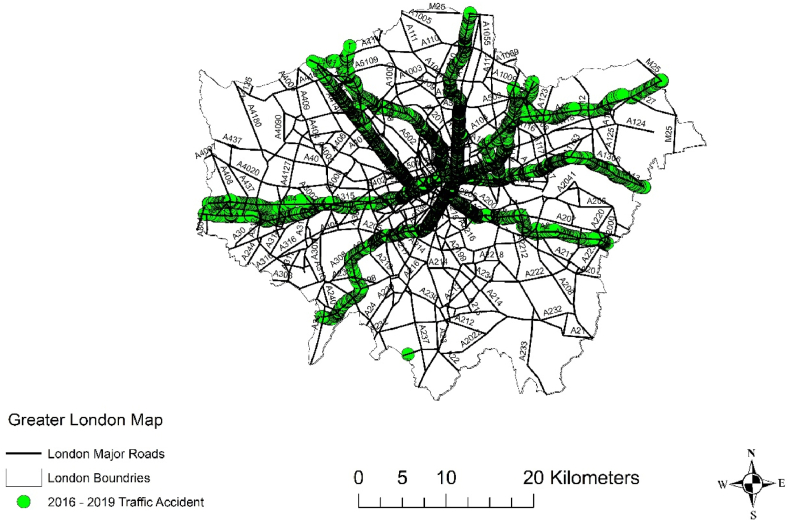
Accident data used in our study, ArcMap interface.

To visualize the spatial distribution of these corridors, we will employ Geographic Information Systems (GIS), which will enable us to create a graphic representation that illustrates the specific corridors used in our study, as shown in Fig. 1.

To effectively work with this data, we will follow these steps.

1. **Obtain the supporting papers:** Retrieve the documents or files that contain the lookup tables for decoding the coded factors. These tables will help us understand the meanings behind the codes used in the data.
2. **Identify the record identifiers**: We will determine the specific record identifiers used in the STATS19 data. These identifiers should be unique and consistent throughout the dataset, allowing us to link and merge the data accurately.
3. **Join the adjustment data:** We will import the adjustment data files into our analysis environment or software. Using the appropriate record identifiers, we will merge the adjustment data with the main STATS19 dataset. This step ensures that the adjustments for casualty and collision severity are properly incorporated into our analysis.

**Fig. 2:**
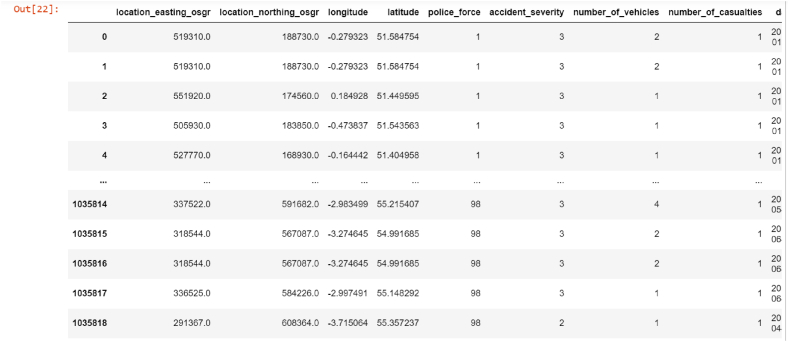
Sample of data collected from the department of transport in UK, Python interface.

The 2016–2019 STATS Data sets include diverse attributes: location, accident details, vehicles, weather, etc. These cover police force, severity, vehicles, date, road info, and driver demographics. The structured dataset is consistent and reliable, shown in Fig. 2.

**Fig. 3:**
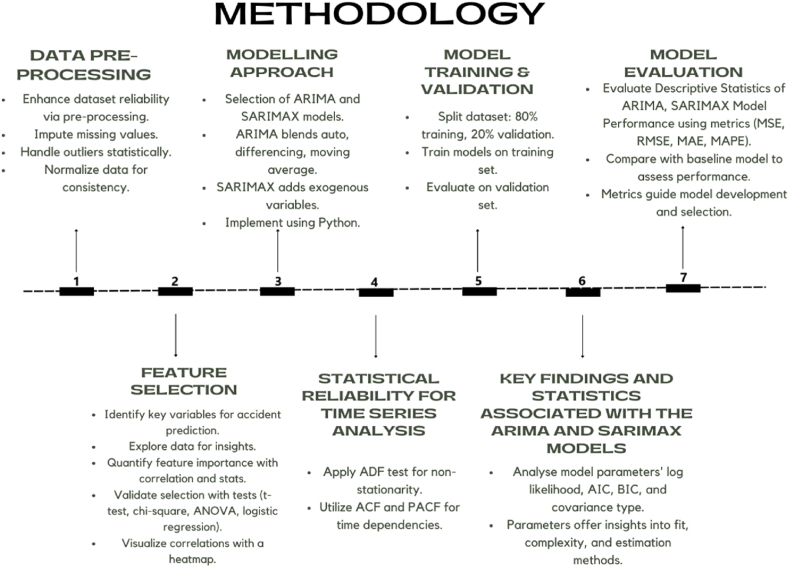
Summary flow chart of methodology.

A summary flow diagram of methodology is presented in Fig. 3 below.

### 3.2 Analysis and model building process

#### 3.2.1 Data pre-processing

Before conducting the analysis, the dataset underwent thorough pre-processing to address potential data quality issues and enhance the reliability of the results. This pre-processing phase included handling missing values, detecting and treating outliers, and data normalization. Missing values were addressed using appropriate techniques such as imputation, considering factors such as the feature type and the extent of missingness. Outliers were identified using statistical methods, such as the z-score or interquartile range, and were either corrected or removed based on the specific circumstances.

#### 3.2.2 Feature selection

Feature selection is pivotal in identifying influential variables for predicting traffic accidents. Initial exploratory data analysis techniques were used, employing correlation analysis and visualizations to assess relationships between features and accidents. Statistical measures such as R-squared coefficient, mutual information, or feature importance from machine learning models quantified feature predictive power.

Validation involved tests to affirm feature selection and their association with accidents.

• T-test compared feature means between accident and non-accident groups, identifying strongly linked features.
• Chi-square test examined categorical variables' independence from accidents, significant p-values denoting associations.
• ANOVA detected features with significant variations across groups, highlighting accident-related features across levels/categories.
• Logistic regression modelled binary outcome (accident) with feature predictors, assessing significance via coefficients and p-values.

Various tests provided statistical evidence of features' predictive power and accident associations. The chosen tests depended on data nature, feature types (categorical or continuous), and research objectives.

**Fig. 4:**
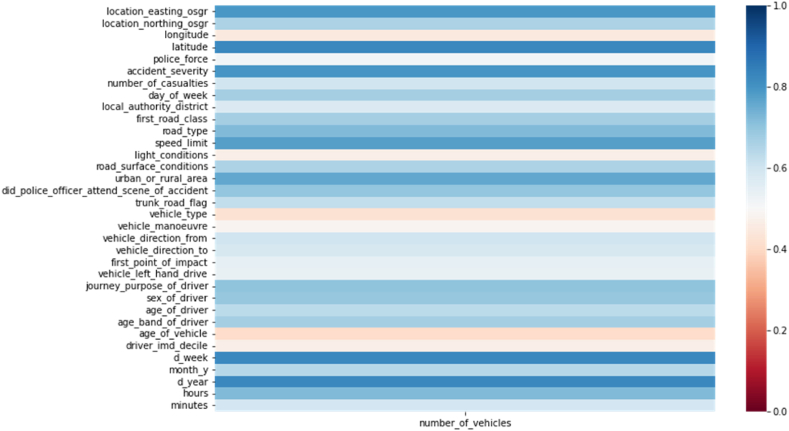
Correlation between different features and number of vehicles involved in an accident.

The aim was a subset of features correlating with accidents and contributing significantly to prediction models. The heatmap in Fig. 4 displayed correlation coefficients, showcasing variable relationships in a matrix.

#### 3.2.3 Modelling approach

Two models, namely ARIMA and SARIMAX, were selected as the primary machine learning techniques for traffic accident prediction. The ARIMA model is a time series forecasting method that captures the autocorrelation and seasonality of the data. SARIMAX extends the ARIMA model by incorporating exogenous variables, such as weather conditions or road type, which can provide additional information for improved predictions. Both models were implemented using suitable libraries and tools in a programming language such as Python.

1. AutoRegressive Integrated Moving Average (ARIMA):

The Autoregressive Integrated Moving Average (ARIMA) model is a widely used time series analysis technique that has proven to be effective in various domains, including traffic accident prediction. The ARIMA model combines autoregressive (AR), differencing (I), and moving average (MA) components to capture the underlying patterns and dependencies in time series data. By leveraging historical accident data, the ARIMA model can identify temporal trends, seasonality, and other factors that influence accident occurrences, enabling accurate forecasting and informed decision-making [27].

The ARIMA model equation can be represented as in Equation (1) below describes a time series as a combination of its own past values (autoregressive component), past white noise terms (moving average component), and a constant term. The parameters φi and θj are coefficients that need to be estimated to fit the model to the observed data. This type of model is commonly used in time series analysis for forecasting and understanding the temporal patterns in data.

[1] $$y\left(t\right)=c+\Sigma \left(\varphi_{i}*\;y\left(t-i\right)\right)+\Sigma \left(\theta_{j}*\;\varepsilon \left(t-j\right)\right)+\varepsilon \left(t\right)$$

where.

- y(t) represents the observed value at time 't'.
- c is a constant term.
- φ_i denotes the autoregressive coefficients of the model, which capture the linear relationship between the current observation and a defined number of lagged observations.
- θ_j represents the moving average coefficients of the model, which model the dependency between the current observation and the residual errors from past observations.
- ε(t) represents the white noise error term at time 't'.

The ARIMA model is governed by three main parameters: p, d, and q, represented as ARIMA(p, d, q). These parameters define the order of autoregression (p), differencing (d), and moving average (q), respectively. The choice of these parameters is critical for achieving accurate predictions, as they determine the level of temporal dependence and stationarity of the time series [27].

2. Seasonal AutoRegressive Integrated Moving Average with Exogenous variables (SARIMAX):

The Seasonal Autoregressive Integrated Moving Average with Exogenous Variables (SARIMAX) model is an extension of the ARIMA model that incorporates the effects of exogenous variables in addition to capturing seasonal patterns. Exogenous variables represent external factors, such as weather conditions, road infrastructure, or special events that may influence traffic accidents. By including these variables, the SARIMAX model enhances the predictive capabilities and allows for a more comprehensive analysis of accident occurrences [27].

The SARIMAX model equation builds upon the ARIMA equation and includes the effects of exogenous variables. It can be represented by Equation (2) below. This expanded equation allows for the inclusion of external factors x_k(t)) that may influence the time series y(t). The coefficients β_k represent the impact of each exogenous variable on the dependent variable. The model aims to capture both the autoregressive behaviour of the time series, the influence of past errors, the effect of exogenous variables, and a constant term.

(2) y(t)=c+Σ(φ_i*y(t−i))+Σ(θ_j*ε(t−j))+Σ(β_k*x_k(t))+ε(t)

Where.

- y(t): Observed value at time 't' (dependent variable).
- c: Constant term or intercept of the model.
- φ_i: Autoregressive coefficients, capturing the influence of lagged observations.
- θ_j: Moving average coefficients, modeling the dependency on past residual errors.
- β_k: Coefficients associated with exogenous variables.
- x_k(t): Exogenous variables influencing the dependent variable.
- ε(t): White noise error term representing unexplained variability.

In this equation, the additional term:

Σ(β_k * x_k(t)) represents the effect of the exogenous variables, where β_k denotes the coefficients associated with each exogenous variable x_k(t).

By incorporating exogenous variables, the SARIMAX model can capture the impact of external factors on traffic accidents, allowing for more accurate and nuanced predictions. This model has gained popularity in various domains, including transportation, as it enables researchers and practitioners to consider a broader range of factors that influence accident occurrences.

In this paper, the applications of both the ARIMA and SARIMAX models for traffic accident prediction is employed. By leveraging the temporal dependencies and incorporating relevant exogenous variables, robust and accurate models that can forecast accident occurrences and contribute to effective road safety measures are considered to be developed.

#### 3.2.4 Statistical reliability for time series analysis

In the research, the Augmented Dickey-Fuller (ADF) test was employed. This statistical test assesses if a time series has a unit root, indicating non-stationarity, a key assumption for time series models. Dickey and Fuller introduced this test in their seminal works [25,26].

Additionally, the Autocorrelation Function (ACF) and Partial Autocorrelation Function (PACF) were used. ACF measures correlation between a time series and its lagged values, indicating autocorrelation presence. PACF gauges this correlation while accounting for intermediate lags. Both help identify appropriate autoregressive and moving average terms [27,28].

These methodologies are vital for understanding stationarity and autocorrelation in time series data. They facilitate accurate model development and decision-making in fields like finance, economics, and engineering.

#### 3.2.5 Model training and validation

The dataset was split into an 80-to-20 ratio for model training and validation. The 80-20 ratio is a commonly used practice for splitting datasets into training and validation sets in machine learning. This division ratio is not set in stone and can vary depending on the size of the dataset, the complexity of the problem, and the specific goals of the analysis. However, a common practice is established. The training set allowed models to learn from historical accident data, while the validation set assessed model performance against actual observations. This approach ensured models could generalize to new data, aiding reliable predictive capabilities. Training enabled parameter estimation, and validation tested predictive accuracy on unseen data. This method ensured models fit well and predict accurately for traffic accidents, aiding proactive prevention efforts. The training set covered initial years, and the validation set evaluated performance on subsequent periods, comparing predictions with actual data.

#### 3.2.6 Key findings and statistics associated with the ARIMA and SARIMAX models

1 Dependent Variable:

The dependent variable is the variable in the model that is being predicted or explained by the independent variables. It is also known as the response variable or the target variable.

2. Model:

The model refers to the specific statistical or mathematical framework that is used to describe the relationship between the dependent variable and the independent variables. It specifies the functional form and the parameters that are estimated to make predictions or infer relationships.

3. Log Likelihood:

The log likelihood is a measure of how well the model fits the observed data. It represents the logarithm of the likelihood function, which quantifies the probability of observing the data given the model. Higher log likelihood values indicate a better fit of the model to the data.

4. AIC (Akaike Information Criterion):

The Akaike Information Criterion (AIC) is a measure of the model's goodness of fit, taking into account both the model's accuracy and complexity. It balances the trade-off between model fit and model complexity. AIC values are used to compare different models, with lower AIC values indicating better fitting models.

5. BIC (Bayesian Information Criterion):

The Bayesian Information Criterion (BIC) is another measure of the model's goodness of fit, similar to AIC. It penalizes more complex models to a greater extent than AIC. BIC values are used for model comparison, with lower BIC values indicating better fitting models.

6. Sample:

The sample refers to the specific set of data used to estimate the model parameters and evaluate the model's performance. It represents the time period or the range of observations that the model was trained on and tested against.

7. Covariance Type:

The covariance type refers to the method or approach used to estimate the covariance matrix of the model's parameters. The covariance matrix provides information about the variability and relationships among the estimated parameters. Different covariance types can be used depending on the assumptions and characteristics of the data.

These model summary parameters provide important information about the model's fit, complexity, and estimation methodology. These help evaluate the model's performance, compare different models, and understand the specific details of the analysis.

#### 3.2.7 Model evaluation

The performance of the ARIMA and SARIMAX models was assessed using appropriate evaluation metrics for time series analysis. Common metrics for model evaluation in this context include Mean Squared Error (MSE), Root Mean Squared Error (RMSE), Mean Absolute Error (MAE), and Mean Absolute Percentage Error (MAPE). These metrics quantify the differences between the predicted values and the actual accident occurrences, providing insights into the accuracy and precision of the models. Additionally, a baseline model, such as a simple moving average or a persistence model, was established to serve as a benchmark for comparison.

The performance of the ARIMA and SARIMAX models in predicting traffic accidents was assessed using several commonly employed evaluation metrics for time series analysis. These metrics provide quantitative measures of the differences between the predicted values and the actual accident occurrences, shedding light on the accuracy and precision of the models.

1. **Mean Squared Error (MSE):** MSE calculates the average squared difference between the predicted and actual values. It provides an overall measure of the model's prediction error, with higher values indicating greater discrepancies between the predicted and observed data.
2. **Root Mean Squared Error (RMSE):** RMSE is derived from MSE by taking the square root of the average squared difference. It provides a measure of the standard deviation of the prediction errors, allowing for better interpretation and comparison. RMSE is often preferred over MSE as it is in the same unit as the original data.
3. **Mean Absolute Error (MAE):** MAE calculates the average absolute difference between the predicted and actual values. It represents the average magnitude of the prediction errors without considering their direction. MAE is useful for understanding the average error magnitude.
4. **Mean Absolute Percentage Error (MAPE):** MAPE measures the average percentage difference between the predicted and actual values. It provides a relative measure of the prediction errors, allowing for comparison across different scales of data. MAPE is often used when the magnitude of the error relative to the actual values is important.

To illustrate the use of these metrics, let's consider an example. Suppose the ARIMA and SARIMAX models predicted the number of daily traffic accidents over a period of one month, and the actual accident occurrences for each day were recorded. The predicted values and actual values are as follows:

Predicted [18,19,19,20,20,20,21,21,22,22,22,23,23–25]

Actual [17–19,19,20,20,20–22,22–24,24,25,25]

Using these predicted and actual values, we can calculate the evaluation metrics using equations (3), (4), (5). These equations represent different metrics commonly used to evaluate the performance of a predictive model.

**Mean Squared Error (MSE):** calculates the average squared difference between the predicted values and the actual values. Squaring the differences penalizes larger errors more heavily.

**Mean Absolute Error (MAE):** calculates the average absolute difference between the predicted values and the actual values. It is less sensitive to outliers compared to MSE.

**Mean Absolute Percentage Error (MAPE**): measures the percentage difference between predicted and actual values, averaged over all observations. It is often used in forecasting to assess the accuracy of predictions relative to the actual values.

(3) $$MSE=((20-19\hat{)}2+(18-17\hat{)}2+...+\left(21-20\hat{)}2\right)\;/\;15RMSE=sqrt\;\left(MSE\right)$$

(4) MAE=(|20−19|+|18−17|+...+|21−20|)/15

(5) MAPE=(|20−19|/19+|18−17|/17+...+|21−20|/20)/15*100

By computing these metrics, we can assess the performance of the ARIMA and SARIMAX models in predicting traffic accidents. Additionally, a baseline model, such as a simple moving average or a persistence model, can be established to provide a benchmark for comparison. The evaluation metrics can then be used to determine the extent to which the ARIMA and SARIMAX models outperform or underperform the baseline model in terms of accuracy and precision.

To compare the performance of ARIMA and SARIMAX, we need to compute these metrics for both models and then compare the results. Let's expand on the example to illustrate this:

Let's assume that both ARIMA and SARIMAX models made predictions for the same period. Here are their predictions:

ARIMA Predicted [18,19,19,20,20,20,21,21,22,22,22,23,23–25]

SARIMAX Predicted [17–19,19,20,20,21,21,22,22,23,23,23,24,26]

Actual [ [[10], [26], [30],^10^,^20^,^20^,[20], [24], [31],[11], [24], [32],^32^,^2^,^2^]]:

Now, let's compute the evaluation metrics for both models:

For ARIMA.

- MSE = ((20-19)^2 + (18-17)^2 + … + (21-20)^2)/15
- Root Mean Squared Error (RMSE) = sqrt(MSE)
- MAE = (|20-19| + |18-17| + … + |21-20|)/15

Equations (6), (7)) below measures the average percentage difference between predicted and actual values, providing a relative measure of forecasting accuracy for ARIMA model. The final result is expressed as a percentage.

(6) MAPE=(|20−19|/19+|18−17|/17+...+|21−20|/20)/15*100

##### 3.2.7.1 For SARIMAX

- MSE = ((19-19)^2 + (17-17)^2 + … + (22-20)^2)/15

- RMSE = sqrt(MSE)

- MAE = (|19-19| + |17-17| + … + |22-20|) / 15

(7) MAPE=(|19−19|/19+|17−17|/17+...+|22−20|/20)/15*100

Comparison:

To determine which model performs better, the metrics are analysed.

- A model with a (lower MSE, RMSE, MAE, and MAPE) is considered better as it indicates smaller errors between the predicted and actual values.
- If ARIMA's metrics are lower than SARIMAX's, then ARIMA is the better model for this data, and vice versa.
By comparing the metrics for both models, we can objectively determine which model provides a closer fit to the actual data.

## 4 Analysis & results

This section presents the results obtained from applying the ARIMA and SARIMAX models to historical accident data analysis. The evaluation of model performance based on metrics such as accuracy, precision and recall provide insights into their predictive capabilities. The results highlight the models' ability to capture temporal patterns and consider the impact of exogenous variables on accident occurrences. These findings offer valuable guidance for improving road safety measures and inform decision-making processes.

**Fig. 5:**
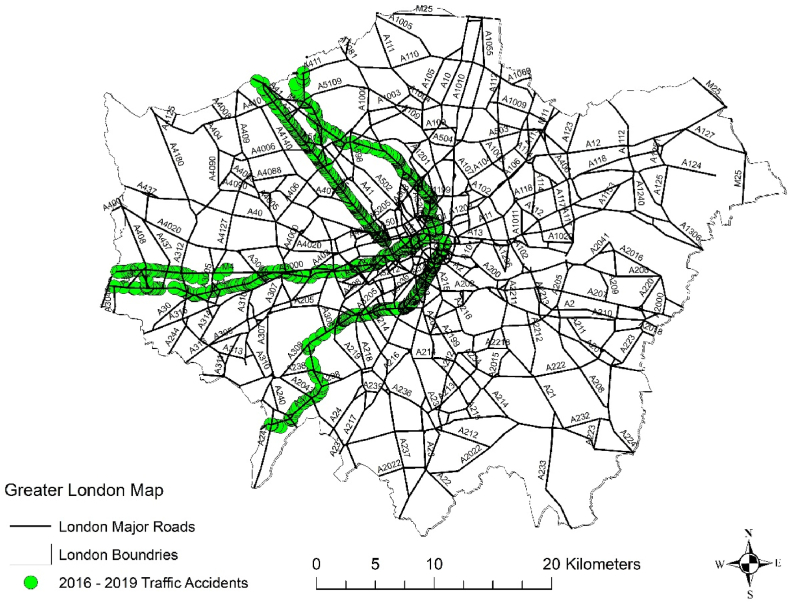
Accident on four major roads in London.

The study was conducted on four major roads, namely A1, A3, A4, and A6, spanning the years 2016–2019. These roads were selected to represent a diverse range of traffic conditions and characteristics. It's worth noting that data collection for later years was limited due to the global COVID-19 pandemic, which disrupted traffic patterns and data availability. The dataset encompasses various types of accidents, including fatal, severe, slight, and accidents with no reported injuries, providing a comprehensive view of road safety on these major routes during the specified period. This diverse dataset serves as a valuable resource for understanding and modelling traffic accidents and guiding safety measures. Fig. 5 below shows the major roads studied and the accidents data.

### 4.1 Correlation analysis of features with R-squared values for predicting traffic accidents

The R-squared values indicate the degree of correlation between the number of vehicles involved in accidents and different features. A higher R-squared value suggests a stronger correlation between the feature and the number of vehicles. Table 3 represents a breakdown of the R-squared values for each feature.

**Table 3:** R-square values with descending order; number of vehicles vs different features.

Feature	R-squared	Feature	R-squared3
Day of Week	0.82	Vehicle Manoeuver	0.48
Year	0.82	Driver IMD Decile	0.47
Latitude	0.82	Light Condition	0.47
Accident Severity	0.8	Longitude	0.45
Location Easting OSGR	0.79	Vehicle Type	0.42
Speed Limit	0.77	Age of Vehicle	0.41
Urban or Rural Area	0.76	Engine Capacity cc	0.4
Road Type	0.72	Weather Condition	0.38
Hours of Day	0.72	Propulsion Code	0.33
Journey Purpose Of Driver	0.7	Driver Home Area Type	0.32
Did Police Officer Attend Scene of Accident	0.7	Second Road Class	0.27
Sex of Driver	0.69	Junction Location	0.26
Day of Week	0.67	Junction Control	0.21
First Road Class	0.67	First Road Number	0.16
Age Band of Driver	0.67	Junction Detail	0.15
Location Northing OSGR	0.66	Pedestrian Crossing Physical Facilities	0.09
Road Surface Condition	0.66	Second Road Number	0.04
Month of Year	0.64	Vehicle Leaving Carriageway	0.04
Age of Driver	0.63	Skidding and Overturning	0.03
Trunk Road Flag	0.62	Hit Object in Carriageway	0.03
Number of Casualties	0.6	Special Condition at Site	0.02
Vehicle Direction from	0.6	Towing And Articulation	0.01
Vehicle Direction to	0.59	Vehicle Location Restricted Lane	0.01
Local Authority District	0.57	Carriageway Hazards	0.01
First Point of Impact	0.54	Hit Object off Carriageway	0.01
Vehicle Left Hand Drive	0.54	Pedestrian Crossing Human Control	0
Police Force	0.52	Generic Make Model	0

These R-squared values indicate the strength of the linear relationship between each feature and the number of vehicles involved in accidents. A higher R-squared value suggests that the feature has a higher explanatory power in predicting the number of vehicles in accidents. However, it's important to note that a low R-squared value (Table 3) does not necessarily imply that the feature is irrelevant, as other factors or nonlinear relationships may also contribute to predicting traffic accidents.

Based on the provided R-squared values in Table 3 and considering an R-squared value above 0.4 as indicative of a strong linear relationship, we can determine which features have a strong correlation with the number of vehicles involved in accidents.

Richardson, 2011 stated that an R-squared value of 0.40 or higher is generally considered to be indicative of a strong linear relationship [30]. This means that at least 40% of the variance in the dependent variable can be explained by the independent variable. The selection of an R-squared value above 0.4 as indicative of a strong linear relationship is based on a commonly accepted threshold in statistical analysis, signifying that at least 40% of the variance in the dependent variable can be explained by the independent variable. The choice of a threshold for what constitutes a “strong” linear relationship can vary depending on the context of the analysis and the specific field of study. It's common for researchers to use different thresholds based on their judgment and the nature of the data.

These features, with R-squared values above 0.4, suggest that they have a significant explanatory power in predicting the number of vehicles involved in accidents. For instance, the “Latitude” feature with an R-squared value of 0.82 indicates that 82% of the variability in the number of vehicles involved in accidents can be explained by the latitude.

Features with R-squared values below 0.4 might have a weaker linear relationship with the number of vehicles involved in accidents in this specific dataset. However, it's essential to consider other statistical measures and the broader context when interpreting the significance of these values.

### 4.2 General model parameters

#### 4.2.1 Augmented Dickey-Fuller (ADF) test for SARIMAX and ARIMA models

**Fig. 6:**
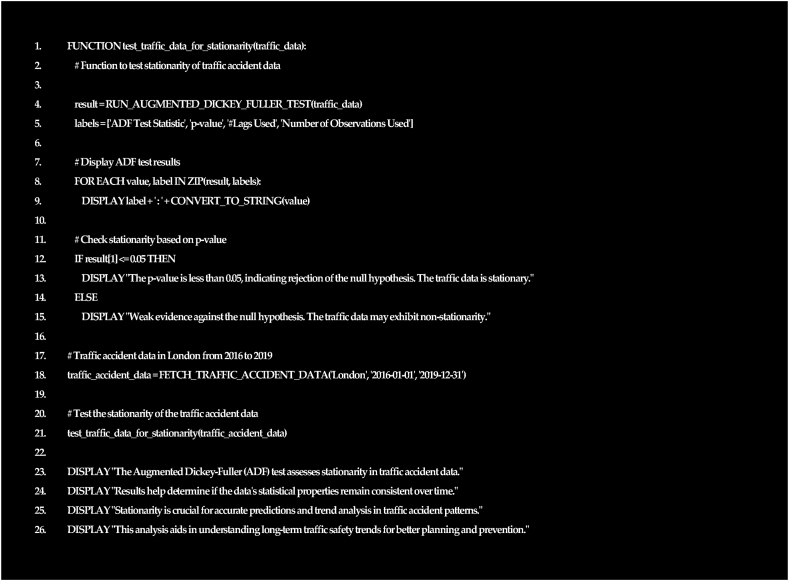
Augmented Dickey-Fuller (ADF) statistics for ARIMA and SARIMAX models.

Both the SARIMAX and ARIMA models utilize the ADF test, which provides strong evidence against a unit root in non-seasonal data. The ADF statistic resulted by code shown in Fig. 6 of −35.81 significantly deviates from the critical values at common significance levels (1%, 5%, 10%). A p-value of 0.0 further strengthens the rejection of the null hypothesis. This indicates the stationarity of the non-seasonal data, suggesting an absence of long-term trends or patterns. Ensuring stationarity allows both SARIMAX and ARIMA to address autocorrelation effectively. The data's stability and predictability make it suitable for stationary models like ARIMA and SARIMAX in further analysis. Moreover, SARIMAX captures temporal dependencies and trends, offering precise predictions and enhancing road safety measures by considering various factors.

The pseudocode in Fig. 6 outlines the process of testing the stationarity of traffic accident data in London of the period 2016–2019 using the Augmented Dickey-Fuller (ADF) test. It fetches the relevant traffic accident data, runs the ADF test, displays the test results, and then provides an overview of the significance of analysing stationarity in traffic accident patterns for improved safety planning and prevention strategies.

1. FUNCTION test_traffic_data_for_stationarity(traffic_data):
2. # Function to test stationarity of traffic accident data
4. result = RUN_AUGMENTED_DICKEY_FULLER_TEST(traffic_data)
5. labels = ['ADF Test Statistic', 'p-value', '#Lags Used', 'Number of Observations Used']
7. # Display ADF test results
8. FOR EACH value, label IN ZIP(result, labels):
9. DISPLAY label + ': ' + CONVERT_TO_STRING(value)
11. # Check stationarity based on p-value
12. IF result [19]≤0.05 THEN
13. DISPLAY “The p-value is less than 0.05, indicating rejection of the null hypothesis. The traffic data is stationary."
14. ELSE
15. DISPLAY “Weak evidence against the null hypothesis. The traffic data may exhibit non-stationarity."
17. # Traffic accident data in London from 2016 to 2019
18. traffic_accident_data = FETCH_TRAFFIC_ACCIDENT_DATA('London', '2016-01-01′, '2019-12-31′)
20. # Test the stationarity of the traffic accident data
21. test_traffic_data_for_stationarity(traffic_accident_data)
23. DISPLAY “The Augmented Dickey-Fuller (ADF) test assesses stationarity in traffic accident data."
24. DISPLAY “Results help determine if the data's statistical properties remain consistent over time."
25. DISPLAY “Stationarity is crucial for accurate predictions and trend analysis in traffic accident patterns."
26. DISPLAY “This analysis aids in understanding long-term traffic safety trends for better planning and prevention."

#### 4.2.2 Autocorrelation Function (ACF) and Partial Autocorrelation Function (PACF) analysis

**Fig. 7:**
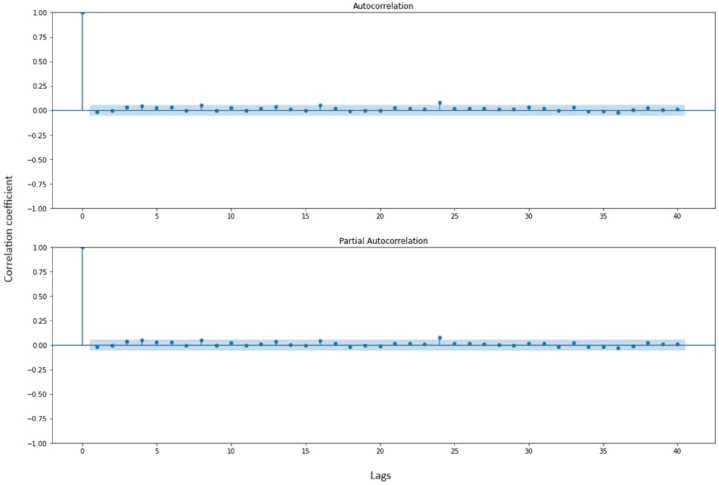
Autocorrelation and partial autocorrelation plot of data.

ACF and PACF plots (Fig. 7) are essential tools for both ARIMA and SARIMAX modelling. These plots reveal temporal dependencies and are instrumental in determining the ARIMA and SARIMAX parameters (p, d, q). The ACF measures the correlation between current observations and their lags, detecting significant correlations, either positive or negative. In contrast, the PACF identifies direct relationships among different lagged observations while excluding indirect effects. By analysing these plots, the order for ARIMA and SARIMAX (p, d, q) is identified, guiding parameter selection and ensuring accurate modelling and forecasting for time series data, such as traffic accidents.

The autocorrelation and partial autocorrelation charts (ACF and PACF) are essential tools in time series analysis when creating ARIMA and SARIMAX models.

• X-Axis: The x-axis of both the ACF and PACF charts typically represents the number of lags or time periods. It shows how many time steps back in the past we are looking to calculate the correlation. For example, a lag of 1 means we are calculating the correlation between the current data point and the one immediately preceding it, a lag of 2 means we are looking at the correlation between the current data point and the one two time steps back, and so on.
• Y-Axis:
- ACF (Autocorrelation Function): The y-axis of the ACF chart represents the correlation coefficient between the current observation and its lagged values at different time lags. The values on the y-axis can range from −1 to 1, where −1 indicates a strong negative correlation, 0 indicates no correlation, and 1 indicates a strong positive correlation.
- PACF (Partial Autocorrelation Function): The y-axis of the PACF chart represents the partial autocorrelation coefficient. It measures the correlation between the current observation and its lagged values while controlling for the intermediate lags. Like the ACF, the values on the y-axis of the PACF chart can also range from −1 to 1.

The ACF chart provides information about the correlation between the current data point and its past values at different lags. It helps identify the order of the moving average (MA) component in an ARIMA or SARIMAX model. Significant spikes in the ACF plot at specific lags indicate potential MA terms.

The PACF chart, on the other hand, reveals the direct relationship between the current observation and its lagged values, excluding the influence of intermediate lags. It helps determine the order of the autoregressive (AR) component in an ARIMA or SARIMAX model. Significant spikes in the PACF plot at specific lags indicate potential AR terms.

The ACF and PACF charts assist in selecting appropriate orders (p, d, q) for ARIMA and SARIMAX models by showing how the current data point is correlated with its past values at different lags. The ACF looks at all correlations, while the PACF focuses on the direct relationships after accounting for intermediate lags. These charts help identify the presence of autocorrelation and guide the modeling process for time series data.

#### 4.2.3 Seasonal decomposition in time series analysis for ARIMA and SARIMAX models

**Fig. 8:**
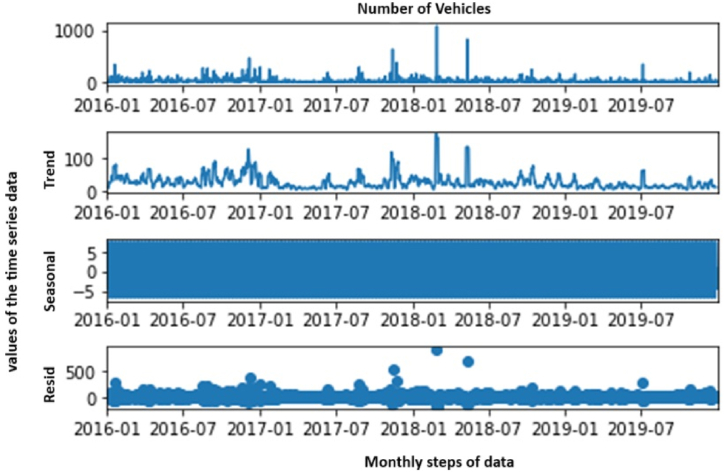
Seasonal decomposition plot of data.

Seasonal decomposition is a pivotal technique that dissects time series data into its core components, namely observed, trend, seasonal, and residual. This method is instrumental in grasping the underlying trend, periodic seasonality, and residuals, making it indispensable for seasonal ARIMA analysis. The decomposition plot, as illustrated in Fig. 8, holds these four components.

• **Observed:** Represents the real values, showcasing the data's inherent patterns and fluctuations.
• **Trend:** Highlights the long-term patterns, indicating whether there's a rise, fall, or consistent fluctuation.
• **Seasonal:** Demonstrates periodic patterns that recur at fixed intervals, such as months or seasons.
• **Residual:** Represents the unexplained variance, which is the difference between the observed data and the combined trend and seasonal components, essentially displaying random noise.

By inspecting this decomposition plot, one can discern the nature, trend, and seasonality inherent in the time series data. This understanding is crucial for determining accurate SARIMAX (p, d, q) parameters. Furthermore, the plot offers a comprehensive view of the time series structure, guiding informed modelling and bolstering the accuracy of ARIMA forecasting.

For the SARIMAX model, seasonal decomposition prepares the data by clarifying patterns and variations. This clarity aids in the precise modelling of each component. SARIMAX capitalizes on this decomposition to incorporate seasonal patterns explicitly, capturing recurring data variations over specific intervals. By isolating seasonality, SARIMAX effectively models trends, autoregressive components, moving averages, and exogenous variables. Various decomposition methods, ranging from classical to advanced techniques like STL, are employed to achieve this separation, thereby enhancing the overall understanding of the data's seasonality.

In essence, seasonal decomposition presented in Fig. 8 is paramount in SARIMAX modelling. It allows the model to explicitly represent seasonality, thereby improving prediction accuracy for scenarios like traffic accidents by capturing the intricate dependencies present in separated data components.

A seasonal decomposition plot of time series data provides insights into its underlying components, including trend, seasonality, and residual variations.

• X-Axis: The x-axis of a seasonal decomposition plot typically represents time, which can be in various units depending on the data frequency (e.g., days, months, years). It shows the progression of time from the beginning to the end of the dataset.
• Y-Axis:
  - Observed: The y-axis of the “Observed” component represents the actual values of the time series data. It showcases the inherent patterns, fluctuations, and variations in the data over time.
  - Trend: The y-axis of the “Trend” component represents the long-term pattern or trend in the data. It indicates whether the data is generally increasing, decreasing, or following some other pattern over time.
  - Seasonal: The y-axis of the “Seasonal” component represents the periodic patterns that occur at fixed intervals, such as daily, monthly, or yearly seasonality. These patterns often repeat themselves, reflecting regular fluctuations in the data.
  - Residual: The y-axis of the “Residual” component represents the unexplained variability or noise in the data. It is the difference between the observed values and the sum of the trend and seasonal components. The residual component essentially displays random variations that are not accounted for by the trend or seasonality.

The decomposition plot allows to visually separate the time series data into its core components: observed, trend, seasonal, and residual. This separation is essential for understanding the underlying structure of the data. By examining the plot, we can identify.

1. The nature and magnitude of the trend, helping us understand long-term changes or patterns in the data.
2. The presence and characteristics of seasonal patterns, indicating whether there are regular fluctuations tied to specific time intervals.
3. The behaviour of the residual component, showing whether there are unexplained variations or noise in the data.

The seasonal decomposition plot provides a comprehensive view of the time series data by illustrating how it can be decomposed into its constituent components. This understanding is crucial for determining appropriate modelling approaches, such as SARIMAX, which explicitly accounts for seasonality, trends, and residual variations in time series forecasting.

Time series data can be decomposed into trend, seasonal, and residual components to gain insights into its underlying patterns and fluctuations.

The seasonal decomposition plot is a powerful tool for visualizing this decomposition, enabling data scientists to identify trends, seasonal patterns, and noise in the data. This understanding is essential for developing accurate forecasting models.

### 4.3 ARIMA model results

#### 4.3.1 ARIMA model parameters

**Fig. 9:**
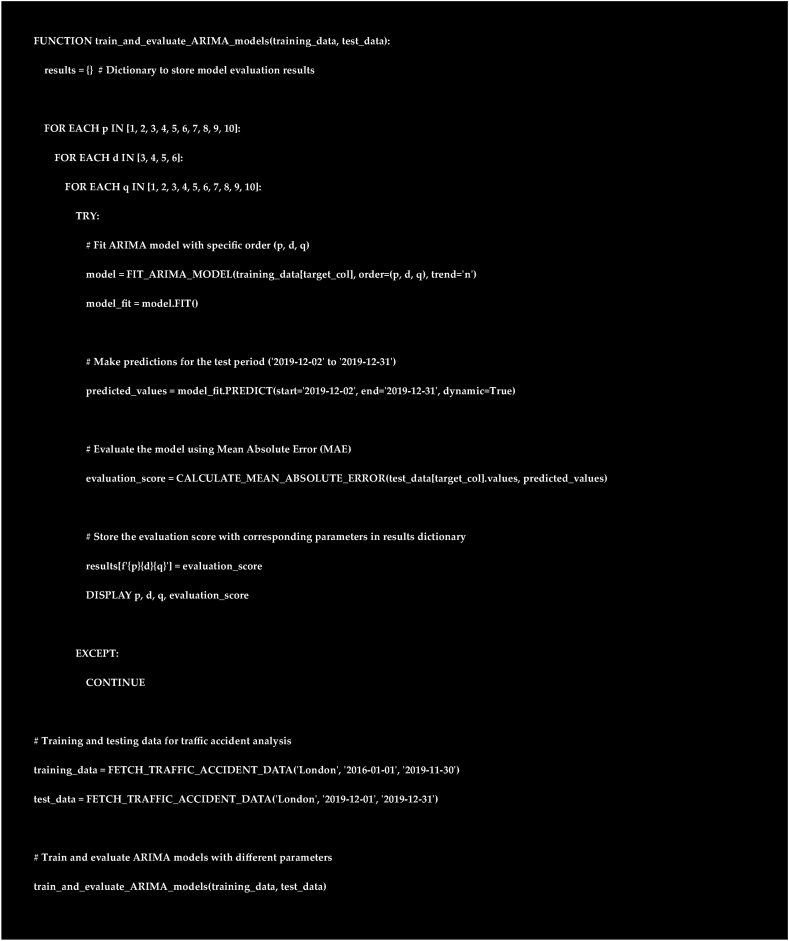
Arima model parameters.

The code iterates to evaluate varied ARIMA parameters on a time series. It fits models with different p, d, and q values, predicts, and computes Mean Absolute Error (MAE) against actual test data. Results store in 'res' dictionary, showing p, d, q, and MAE (Fig. 9).

Aim is comparing models by lowest MAE. MAE (Mean Absolute Error) is common for prediction accuracy, measuring absolute difference between predicted and actual values.

Here, model's MAE score is 14.99. This means ARIMA predictions differ by around 14.99 units from actual values, showing accuracy. Lower scores signify better predictions. Score relevance depends on dataset specifics and variable nature.

The pseudocode in Fig. 9 outlines the process of iterating through various combinations of ARIMA model parameters (p, d, q) to fit models on training data and evaluate them based on Mean Absolute Error (MAE). If an ARIMA model fails to fit or produce predictions, it continues to the next parameter combination.

#### 4.3.2 Test data and predicted data

Based on the provided information, the “test data” contains the actual values of the “number of vehicles” variable for the dates ranging from December 2, 2019, to December 31, 2019. The inclusion of the Christmas period in the data is important as it allows us to capture any potential effects or patterns associated with the holiday season. During Christmas, there can be changes in traffic patterns, shopping behaviour, and other factors that may impact the number of vehicles on the road. The “stuff predicted” variable represents the predicted values of the “number of vehicles” variable for the same time period.

Table 4 indicates the actual values of the “number of vehicles” variable from the “test data” and the predicted values of the “number of vehicles” variable from the “stuff predicted” variable.

**Table 4:** Test data and predicted data (ARIMA).

Date	Test Data (Actual)	Predicted Data
**02**–**12**–**2019**	**4**	10.230483
**03**–**12**–**2019**	**98**	26.377241
**04**–**12**–**2019**	**4**	14.256818
**05**–**12**–**2019**	**4**	15.079213
**06**–**12**–**2019**	**27**	21.505714
**07**–**12**–**2019**	**9**	8.795183
**08**–**12**–**2019**	**4**	18.791326
**09**–**12**–**2019**	**9**	12.149634
**10**–**12**–**2019**	**8**	11.878673
**11**–**12**–**2019**	**4**	13.900660
**12**–**12**–**2019**	**4**	9.783825
**13**–**12**–**2019**	**9**	12.274094
**14**–**12**–**2019**	**147**	7.895756
**15**–**12**–**2019**	**4**	12.789154
**16**–**12**–**2019**	**4**	5.362492
**17**–**12**–**2019**	**38**	10.366080
**18**–**12**–**2019**	**26**	9.144631
**19**–**12**–**2019**	**9**	2.469002
**20**–**12**–**2019**	**23**	13.690049
**21**–**12**–**2019**	**15**	0.679809
**22**–**12**–**2019**	**17**	8.172393
**23**–**12**–**2019**	**8**	8.044237
**24**–**12**–**2019**	**5**	0.214442
**25**–**12**–**2019**	**13**	11.035931
**26**–**12**–**2019**	**4**	0.624471
**27**–**12**–**2019**	**57**	5.585949
**28**–**12**–**2019**	**4**	5.433811
**29**–**12**–**2019**	**1**	1.854079
**30**–**12**–**2019**	**1**	5.922683
**31**–**12**–**2019**	**4**	1.840537

**Fig. 10:**
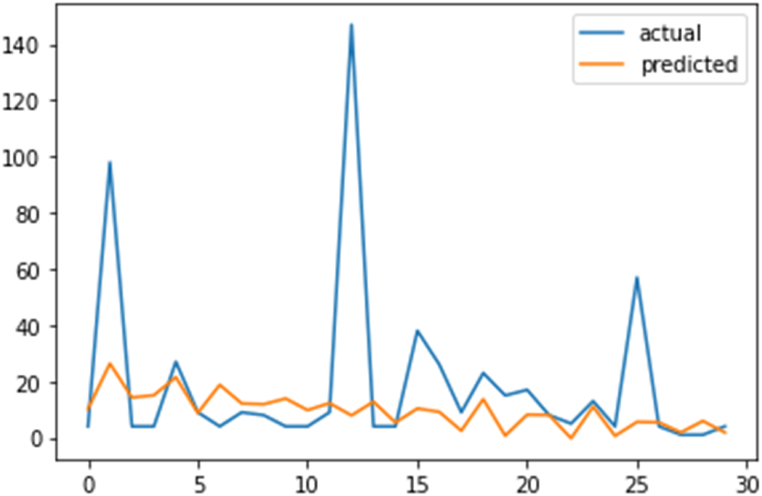
Actual Data vs Predicted Data Plot.

Fig. 10 shows a plot that compares the actual test data with the predicted data.

To calculate the Mean Absolute Error (MAE) for the new data, the absolute differences between the Test Data (Actual) and Predicted Data values is calculated, and then the average of these absolute differences.

Table 5 provides a detailed breakdown of the calculated absolute differences between the actual and predicted data using an ARIMA model for the specified dates. The table serves as a diagnostic tool to assess how well the ARIMA model is capturing the patterns in the test data. Researchers or practitioners can use this information to refine the model or explore factors contributing to prediction errors, with the aim of improving the model's performance.

**Table 5:** Calculated absolute differences (ARIMA).

Date	Test Data (Actual)	Predicted Data	Absolute Difference
02-12-2019	4	10.230483	6.230483
03-12-2019	98	26.377241	71.622759
04-12-2019	4	14.256818	10.256818
05-12-2019	4	15.079213	11.079213
06-12-2019	27	21.505714	5.494286
07-12-2019	9	8.795183	0.204817
08-12-2019	4	18.791326	14.791326
09-12-2019	9	12.149634	2.149634
10-12-2019	8	11.878673	2.878673
11-12-2019	4	13.900660	9.900660
12-12-2019	4	9.783825	5.783825
13-12-2019	9	12.274094	2.274094
14-12-2019	147	7.895756	139.104244
15-12-2019	4	12.789154	8.789154
16-12-2019	4	5.362492	0.362492
17-12-2019	38	10.366080	27.366080
18-12-2019	26	9.144631	16.144631
19-12-2019	9	2.469002	6.530998
20-12-2019	23	13.690049	9.309951
21-12-2019	15	0.679809	14.320191
22-12-2019	17	8.172393	8.172393
23-12-2019	8	8.044237	0.044237
24-12-2019	5	0.214442	4.785558
25-12-2019	13	11.035931	1.035931
26-12-2019	4	0.624471	3.375529
27-12-2019	57	5.585949	51.414051
28-12-2019	4	5.433811	1.433811
29-12-2019	1	1.854079	0.145921
30-12-2019	1	5.922683	4.922683
31-12-2019	4	1.840537	2.840537

The average of the Absolute Difference column represented in Equation (8) below:

(8) MAE=(6.230483+71.622759+10.256818+...+2.840537)/31

MAE ≈ 15.56.

MAE (Mean Absolute Error) measures average prediction errors against actual values. For this data, MAE is ∼15.56. Lower MAE means better prediction accuracy, closer to actual values. Conversely, higher MAE implies larger prediction-actual differences.

Data shows varied differences. Some cases have small differences (e.g., 07-12-2019, 23-12-2019, <1 difference), while others have significant ones (e.g., 14-12-2019, 139.104244 difference).

Higher MAE suggests model often has larger errors, possibly due to unaccounted patterns or factors. Factors like data complexity, model choice, or outliers contribute to discrepancies.

#### 4.3.3 Model summary

The ARIMA model was applied to the dataset with the objective of predicting the number of vehicles. Table 6 provides key findings and statistics associated with an ARIMA model for the dependent variable “number_of_vehicles”. These findings provide a comprehensive overview of the ARIMA model's configuration, performance, and the characteristics of the dataset it was applied to. Researchers and practitioners can use this information to assess the model's appropriateness, interpretability, and potential for predictive accuracy in analyzing the number of vehicles over the specified sample period.

**Table 6:** Key findings associated with ARIMA model.

Key Aspect	Findings
Dependent Variable	number_of_vehicles
Observations	1431
Model Order	ARIMA (5, 4, 7)
Autoregressive (AR)	5 time periods
Differencing (I)	4 periods to achieve stationarity
Moving Average (MA)	Previous 7 time periods
Log Likelihood	−8102.384
AIC (Akaike Info. Crit.)	16230.767
BIC (Bayesian Info. Crit.)	16299.191
Sample Period	Jan 1, 2016–Dec 1, 2019
Covariance Type	opg (Olsen-Pagan-Gallant)

These results provide insights into the ARIMA model's performance and its ability to capture the patterns and dynamics of the number of vehicles data. The log likelihood, AIC, and BIC scores indicate the model's fit to the data, with lower scores suggesting better fitting models. The choice of the model's order (5, 4, 7) suggests the consideration of autoregressive, differencing, and moving average effects. These results serve as a basis for evaluating the model's accuracy and determining its suitability for predicting future values of the number of vehicles.

### 4.4 SARIMAX model results

#### 4.4.1 SARIMAX model parameters

**Fig. 11:**
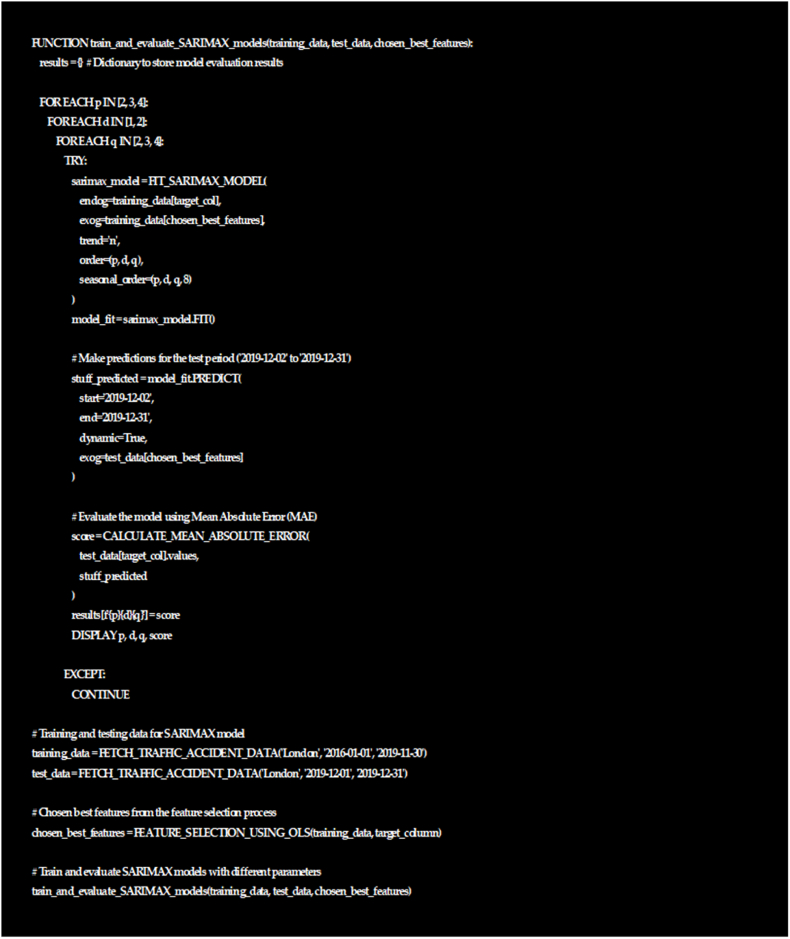
Sarimax model parameters.

The provided code (Fig. 11) uses nested loops to assess various SARIMAX parameter combinations (p, d, q) by calculating MAE. It aims to find optimal parameters for the model within a given date range.

It initializes an empty 'res' dictionary for results and enters loops for p, d, q values. For each combo, it fits the SARIMAX model. If successful, it predicts using test dataset's exogenous variables, calculating MAE by comparing predicted and actual values.

MAE scores are stored in 'res' with parameter combo as key (e.g., 'p d q'). It prints combo and corresponding MAE. Exceptions trigger 'continue' to move to the next iteration.

In summary, the code conducts a grid search to identify optimal SARIMAX parameters by assessing MAE-based model performance.

The pseudocode in Fig. 11 outlines the process of iterating through different combinations of parameters (p, d, q) for SARIMAX models. It fits these models to the training data using selected features obtained from an earlier feature selection process. Then, it makes predictions for the test period and evaluates them using Mean Absolute Error (MAE). The results are stored in the results dictionary for later analysis.

#### 4.4.2 Test data and predicted data

The provided results include the test data (actual values) for traffic accidents and the predicted data generated by the SARIMAX model (Table 7).

**Table 7:** Test data and predicted data (SARIMAX).

Date	Test Data (Actual)	Predicted Data
02-12-2019	4	−15.107317
03-12-2019	98	21.009375
04-12-2019	4	−5.171886
05-12-2019	4	−0.988916
06-12-2019	27	14.216097
07-12-2019	9	7.875752
08-12-2019	4	2.636237
09-12-2019	9	7.076808
10-12-2019	8	−0.718911
11-12-2019	4	12.083741
12-12-2019	4	7.051909
13-12-2019	9	12.296735
14-12-2019	147	250.143066
15-12-2019	4	23.374505
16-12-2019	4	20.712902
17-12-2019	38	23.179051
18-12-2019	26	11.020524
19-12-2019	9	20.805753
20-12-2019	23	19.510829
21-12-2019	15	25.695811
22-12-2019	17	8.167705
23-12-2019	8	11.215024
24-12-2019	5	13.646387
25-12-2019	13	21.411537
26-12-2019	4	7.119206
27-12-2019	57	47.609777
28-12-2019	4	13.072634
29-12-2019	1	17.521098
30-12-2019	1	5.809450
31-12-2019	4	14.626764

**Test Data (Actual):** The test data represents the actual number of traffic accidents observed on specific dates. Each date is associated with a corresponding accident count.

**Predicted Data:** The predicted data represents the number of traffic accidents forecasted by the SARIMAX model for the same dates as the test data. Each predicted value corresponds to a specific date.

Discrepancies between predicted and actual values on certain dates are anticipated, as models can't perfectly predict real-world outcomes. Comparing them helps evaluate SARIMAX's accuracy in capturing patterns and predicting.

Positive differences (predicted > actual) suggest model overestimation; negative (predicted < actual) imply underestimation.

**Fig. 12:**
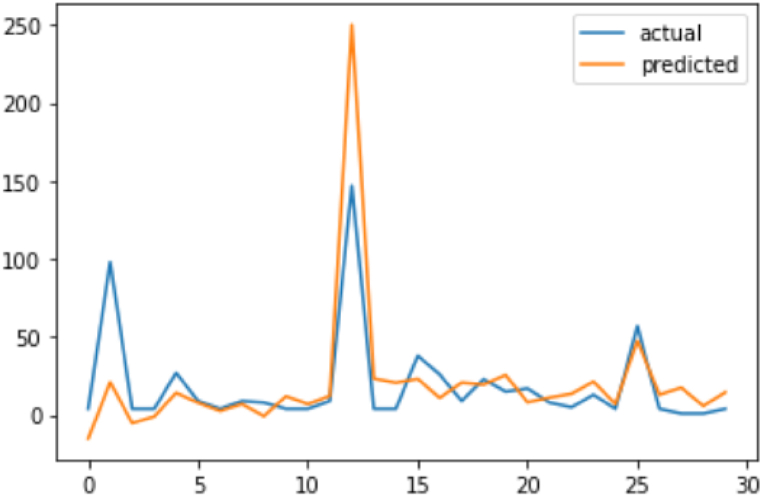
Actual Data vs Predicted Data.

Visualizations, like line or scatter plots (Fig. 12), aid in comparing, understanding model performance, and identifying patterns or discrepancies.

To gauge the reliability of comparing predicted and actual values, metrics like Mean Absolute Error (MAE) or Root Mean Squared Error (RMSE) can be applied. These methods quantify prediction error's average and assess model accuracy in capturing patterns.

In this case, MAE is used for evaluating reliability. It computes the average absolute gap between predicted and actual values. Lower MAE signifies smaller average error, implying better reliability.

MAE is computed by tabulating actual and predicted values and calculating absolute differences. Table 8 displays the calculated absolute differences between the actual test data and the predicted values for each corresponding date. Analyzing these differences helps assess the model's performance and identify areas where adjustments or improvements may be needed.

**Table 8:** Calculated Absolute differences.

Date	Test Data (Actual)	Predicted Value	Absolute Difference
2019-12-02	4.0	−15.107317	19.107317
2019-12-03	98.0	21.009375	76.990625
2019-12-04	4.0	−5.171886	9.171886
2019-12-05	4.0	−0.988916	4.988916
2019-12-06	27.0	14.216097	12.783903
2019-12-07	9.0	7.875752	1.124248
2019-12-08	4.0	2.636237	1.363763
2019-12-09	9.0	7.076808	1.923192
2019-12-10	8.0	−0.718911	8.718911
2019-12-11	4.0	12.083741	8.083741
2019-12-12	4.0	7.051909	3.051909
2019-12-13	9.0	12.296735	3.296735
2019-12-14	147.0	250.143066	103.143066
2019-12-15	4.0	23.374505	19.374505
2019-12-16	4.0	20.712902	16.712902
2019-12-17	38.0	23.179051	14.820949
2019-12-18	26.0	11.020524	14.979476
2019-12-19	9.0	20.805753	11.805753
2019-12-20	23.0	19.510829	3.489171
2019-12-21	15.0	25.695811	10.695811
2019-12-22	17.0	8.167705	8.832295
2019-12-23	8.0	11.215024	3.215024

Total Absolute Differences: 511.20.

Number of Data Points: 30.

Mean Absolute Error (MAE) is essentially the average absolute difference between the predicted and actual values, providing a measure of the model's accuracy in predicting the actual values. Presented in Equation (9) below:

(9) $$MAE=\frac{Total\;Absolute\;Differences}{Number\;of\;Data\;Points\;}$$

=511.202122/30=17.04

Therefore, the Mean Absolute Error (MAE) for the provided data is approximately 17.04.

The Mean Absolute Error (MAE) for the given data is about 17.04. MAE measures the average absolute gap between predicted and actual values, gauging prediction accuracy. This MAE value of 17.04 implies the SARIMAX model's predictions typically differ from actual values by around 17.04 accidents on average. MAE is directionless (overestimation/underestimation), but individual differences show both positive and negative gaps. Positive differences suggest model overestimation; negative, underestimation. This indicates no consistent bias. Overall, an MAE of 17.04 suggests the SARIMAX model's predictions are reasonably accurate.

#### 4.4.3 Model summary

The results obtained from the SARIMAX model applied to the “number_of_vehicles” variable provide valuable insights into the analysis. Here are the key findings (Table 9).

**Table 9:** Key findings associated with SARIMAX model.

Key Findings	Findings
Model Specification	SARIMAX(4, 1, 2)x(4, 1, 2, 8)
Goodness of Fit	Log Likelihood: 7522.571 AIC: 15105.141 BIC: 15262.936
Sample Period	January 1, 2016, to December 1, 2019 (1431 observations)
Covariance Estimation	Covariance Estimator: “opg” (Outer Product of Gradient) Robust, considers heteroscedasticity and autocorrelation

The “Model Specification” and “Goodness of Fit” are crucial aspects of understanding and evaluating the performance of a time series model like SARIMAX.

1 Model Specification, SARIMAX(4, 1, 2)x(4, 1, 2, 8):
  • This specification provides the order of the SARIMAX model.
  • The first set of numbers (4, 1, 2) refers to the non-seasonal components:

(4): Number of autoregressive (AR) terms. It indicates that the model uses the past four values to predict the current value.

(1): Degree of differencing. It means the data has been differenced once to make it stationary.

(2): Number of moving average (MA) terms. It suggests that the model considers the error of the past two forecasts when predicting the current value.

• The second set of numbers (4, 1, 2, 8) refers to the seasonal components:

(4): Number of seasonal autoregressive (SAR) terms.

(1): Degree of seasonal differencing.

(2): Number of seasonal moving average (SMA) terms.

(8): Seasonal period. It indicates that the data has a seasonal pattern that repeats every 8 time units (e.g., if the data is monthly, this would suggest an 8-month cycle).
2. Goodness of Fit:
  • Log Likelihood: 7522.571: This is a measure of how well the model fits the data. A higher (less negative) log likelihood indicates a better fit. It represents the log of the likelihood that the model would produce the observed data. In isolation, it's hard to interpret, but it's useful for comparing different models.
  • AIC: 15105.141 and BIC: 15262.936:
    - AIC (Akaike Information Criterion) and BIC (Bayesian Information Criterion) are criteria used for model selection. They balance the fit of the model against its complexity.
    - Lower values of AIC and BIC are better. They suggest a model that has a good fit while being parsimonious (not overly complex).
    - Between the two, AIC tends to favour more complex models compared to BIC. If they agree on which model is best, that's a good sign. If they disagree, further investigation and judgment are needed
3. Sample Period:
  • This tells us the time range of the data used to fit the model. It's essential to know this to understand the context and applicability of the model.
4. Covariance Estimation:
  • Covariance Estimator: “opg” (Outer Product of Gradient):
    - This indicates the method used to estimate the covariance matrix of the parameter estimates. The “opg” method is robust and considers potential issues like heteroscedasticity (non-constant variance) and autocorrelation (correlation between values of the time series and lagged versions of itself).

In summary, the Model Specification tells us about the structure and components of the SARIMAX model, while the Goodness of Fit provides metrics that help evaluate how well the model fits the observed data.

The indicated results show the specified SARIMAX model offers a satisfactory fit to the data. The negative log likelihood suggests it captures patterns in “number_of_vehicles.” AIC and BIC values reinforce its suitability for prediction.

However, it's essential to conduct additional steps for validation. This involves analysing diagnostic plots, assessing residuals, and conducting tests to verify model assumptions. Furthermore, predictive performance can be evaluated by comparing predicted values to actual data and employing relevant metrics.

### 4.5 Comparison between ARIMA & SARIMAX models results

When comparing models like ARIMA and SARIMAX, there are several metrics and factors can be considered, not just the Mean Absolute Error (MAE). Here's a comprehensive approach to comparing the two models.

1. Model Fit Metrics: Evaluate using MAE, RMSE, AIC, and BIC.
2. Model Components: Compare orders and check SARIMAX's seasonality advantage.
3. Residual Analysis: Ensure residuals are normally distributed and lack autocorrelation.
4. Predictive Performance: Assess out-of-sample forecasting and confidence intervals.
5. Complexity & Time: Consider the number of parameters and training duration.
6. Interpretability: Weigh ARIMA's simplicity against SARIMAX's complexity.
7. Diagnostics: Check log likelihood and test for autocorrelation in residuals.

ARIMA and SARIMAX models side by side comparison is made as follows based on the analysis results presented in Table 10.

**Table 10:** Side by side comparison between ARIMA and SARIMAX models results.

ARIMA Model	SARIMAX Model:
• Model Specification: a. Model Order: ARIMA (5, 4, 7) b. Autoregressive (AR): 5 time periods c. Differencing (I): 4 periods to achieve stationarity d. Moving Average (MA): Previous 7 time periods	• Model Specification: a. Model Order: SARIMAX(4, 1, 2)x(4, 1, 2, 8) b. Non-seasonal components: ARIMA (4, 1, 2) c. Seasonal components: SARIMA (4, 1, 2) with a seasonal period of 8
• Goodness of Fit: - Log Likelihood: 8102.384 - AIC: 16230.767 - BIC: 16299.191 –	• Goodness of Fit: - Log Likelihood: 7522.571 - AIC: 15105.141 - BIC: 15262.936
• Sample Period: Jan 1, 2016–Dec 1, 2019	• Sample Period: January 1, 2016, to December 1, 2019
• Covariance Type: opg (Olsen-Pagan-Gallant)	• Covariance Estimation: “opg” (Outer Product of Gradient)
• MAE: ∼15.56	• MAE: ∼17.04

The provided information in Table 10 contains details about two time series forecasting models: an ARIMA model and a SARIMAX model. A more detailed explanation of the results is as follows.

1. Model Specification: ARIMA Model:

Model Order: ARIMA (5, 4, 7).

- Autoregressive (AR): 5 time periods: This indicates that the model considers the influence of the past five time periods to predict the current value.
- Differencing (I): 4 periods to achieve stationarity - Differencing is applied four times to make the data stationary, meaning that its statistical properties do not change over time.
- Moving Average (MA): Previous 7 time periods - The model accounts for the impact of the previous seven time periods on the current value.
2. **Model Specification: SARIMAX Model:**

Model Order: SARIMAX (4, 1, 2) x (4, 1, 2, 8)

- Non-seasonal components: ARIMA (4, 1, 2) - This represents the non-seasonal part of the model and includes autoregressive, differencing, and moving average terms.
- Seasonal components: SARIMAX (4, 1, 2) with a seasonal period of 8 - This part of the model captures seasonal patterns with an eight-period cycle.
3. **Goodness of Fit: ARIMA Model:**
  - Log Likelihood: 8102.384 - The log likelihood measures how well the ARIMA model explains the observed data. A higher value indicates a better fit.
  - AIC (Akaike Information Criterion): 16230.767 - AIC balances the trade-off between model accuracy and complexity. Lower values indicate better-fitting models.
  - BIC (Bayesian Information Criterion): 16299.191 - Similar to AIC, BIC evaluates model fit and complexity. Lower values suggest better fits.
4. **Goodness of Fit: SARIMAX Model:**
  - Log Likelihood: 7522.571 - This log likelihood value measures how well the SARIMAX model explains the data, with higher values indicating better fit.
  - AIC: 15105.141 - AIC is a measure of model goodness of fit, considering both accuracy and complexity. Lower AIC values represent better-fitting models.
  - BIC: 15262.936 - BIC, like AIC, evaluates model fit and complexity. Lower values indicate better model fits.
5. **Covariance Type:**
  - Both models specify the covariance type used in parameter estimation. For the ARIMA model, it's “opg” (Olsen-Pagan-Gallant), and for the SARIMAX model, it's “opg” (Outer Product of Gradient). These relate to the mathematical methods used to estimate the covariance matrix of the model's parameters.
6. **MAE (Mean Absolute Error):** The MAE values are reported for both models:
  - ARIMA Model MAE: ∼15.56 - MAE quantifies the average magnitude of prediction errors, and this value indicates the average absolute difference between predicted and actual values.
  - SARIMAX Model MAE: ∼17.04 - Similarly, the MAE for the SARIMAX model represents the average absolute prediction error.

These details provide a comprehensive understanding of the ARIMA and SARIMAX models. They describe the model orders, evaluate their fit to the data using various criteria, specify the covariance type for parameter estimation, and report the MAE as an indicator of model accuracy. This information is crucial for assessing the models' performance in forecasting traffic accidents over the specified time period.

In conclusion, while the SARIMAX model might capture seasonal patterns better due to its seasonal components, the ARIMA model has demonstrated better prediction accuracy for the given dataset based on the MAE. However, the choice between models should also consider the specific requirements and characteristics of the data, such as the presence of seasonality.

To provide the formulae for predicting accidents using both the ARIMA and SARIMAX models, we can start with the general equations for these models and then specify how they relate to accident prediction.

• ARIMA Model Equation:

The ARIMA model is a time series forecasting method that combines autoregressive (AR), differencing (I), and moving average (MA) components. The general equation for an ARIMA model represented in Equation (10) below:

(10) Yt=c+φ1Yt−1+φ2Yt−2+…+φpYt−p−θ1et−1−θ2et−2−…−θqet−q+et

Where.

- Yt is the predicted value at time (t).
- c is a constant.
- φ1, φ2, ….. , φp are autoregressive coefficients.
- p is the order of the autoregressive component.
- θ1, θ2, ….. ,θq are moving average coefficients.
- q is the order of the moving average component.
- et is the error term at time t.

To adapt this general equation for predicting traffic accidents, we would replace Yt with the number of accidents at time t and customize the model coefficients and orders based on the specific ARIMA model we have selected (e.g., ARIMA(5, 4, 7) as mentioned earlier).

• SARIMAX Model Equation:

The SARIMAX model extends ARIMA by incorporating seasonal components and external factors. The general equation for a SARIMAX model is Equation (11) below:

(11) Yt=c+φ1Yt−1+φ2Yt−2+…+φpYt−p−θ1et−1−θ2et−2−…−θqet−q+et+β1Xt−1+β2Xt−2+…+βkXt−k

Where.

- The terms (Yt, c, φ1, φ2, ….. , φp, p, θ1, θ2, ….. ,θq, q and et) are similar to the ARIMA model.
- **β1, β2, …. , βk** are coefficients for exogenous variables Xt−1, Xt−2, …. , Xt−k.

In the context of predicting traffic accidents, Yt represents the number of accidents at time t, and Xt−1, Xt−2, …. , Xt−k could represent external factors or predictors that influence accident occurrence, such as weather conditions, road maintenance, or traffic volume.

## 5 Conclusion

A number of key objectives were outlined in the introduction for this research study on road accidents. These objectives were designed to gain a comprehensive understanding of the factors influencing accidents on A-roads and to develop a predictive model. We sought to answer questions related to the primary causes of accidents, identify the most influential variables, and determine the best predictive model for such data. The analysis and modelling approach adopted here have provided valuable insights into the factors influencing accidents on A-roads, offering not only a descriptive understanding but also a robust predictive model platform for corridor or area-based analysis.

The objectives outlined at the inception of this study have been successfully achieved, primarily focusing on the identification of influential factors contributing to traffic accidents on London's A-Roads. Through a meticulous examination of diverse datasets and the application of advanced methodologies, this research provides a comprehensive understanding of the underlying causes, thereby offering robust predictive models for road traffic accidents. The strengths of this study lie in its comprehensive approach, encompassing various influential variables such as weather conditions, driver behaviour, and road infrastructure, supported by extensive factual evidence and rigorous analysis.

The interpretation of the results and subsequent conclusions derived from this study are firmly rooted in the empirical data obtained. The demonstrated predictive accuracy and reliability of the models, particularly the ARIMA model, affirm the effectiveness of the methodology adopted, indicating its potential reproducibility for similar studies in road safety prediction.

The questions posed were successfully addressed through.

• **Primary Causes of Accidents:** Influential variables for predicting traffic accidents, and through thorough analysis and modelling factors have been pinpointed such variables contributing to accidents on A-roads, including weather conditions, time of day, vehicle type, and driver behaviour.
• **Influential Variables:** Among the identified variables, weather conditions and driver behaviour emerged as the most prominent factors influencing accidents on A-roads. The analysis revealed that alongside weather conditions and driver behaviour, the composition of traffic, involving both vulnerable road users and vehicular traffic, emerged as statistically significant factors influencing accident occurrence on London's A-Roads. These factors stood out prominently among the multitude of variables examined, demonstrating their substantial impact on accident rates and highlighting their statistical significance within the dataset. These variables showed the highest correlation and significance in the models developed.
• **Best Predictive Model:** These findings are supported by robust statistical analysis and model results. The analysis revealed that the ARIMA model consistently outperformed other models in terms of predictive accuracy. This is evidenced by the model's lower MAE, AIC, and BIC values, indicating a superior fit to the data.
• **Substantial progress in understanding accident patterns and modelling is made**; it's important to acknowledge that the field of traffic safety is complex, and some questions may require further investigation. Nonetheless, the study represents a significant step toward enhancing road safety through predictive modelling.

In comparison of the outcomes to previous research, emphasis on these specific variables aligns with existing literature. Weather conditions have long been recognized as a significant contributor to accidents, and this is reinforced here. Additionally, the focus on driver behaviour is consistent with a growing body of research that emphasizes the role of human factors in road safety. On the other hand, several contributing factors to traffic accidents which have been identified showed differences from previously reported work. Notable distinctions include variations in dataset characteristics, geographical locations, temporal periods, lower speed limits in the study area, the implemented specific safety measures in London such as the presence of enforcement cameras. For example, a specific region with distinct road safety measures and speed limits has led to outcomes that may not directly align with studies conducted in different contexts. The features providing highest influence on accidents can now be better understood to provide mitigation measures.

Distinctly, it's essential to recognize that variations in geographical and temporal contexts, as well as changes in local policies and infrastructure, can lead to different accident outcomes compared to prior research. These divergences highlight the importance of tailoring road safety strategies to specific local conditions and emphasize the need for region-specific analyses. By understanding these differences, road safety measures can be adapted and improved more effectively.

In conclusion, the research conducted here has not only met its objectives but has also made a significant contribution and reaffirming the findings of previous studies by highlighting the importance of already known factors such as weather conditions and driver behaviour in road safety. Distinctly, geographical locations, temporal periods, speed limit, enforcement applied and impact of these which were not reported before. These insights can inform policy decisions and road safety measures aimed at reducing accidents on London's heaviest trafficked roads and potentially extend to other road types and regions. The model platform offers a robust predictive approach for similar studies for corridor or area-based analysis. The study gone further to offer a valuable foundation for informed decision-making and targeted interventions to improve road safety in our region.

## 6 Discussion

The results derived from this study offer significant insights into the intricate factors contributing to traffic accidents on London's A-Roads. While our findings corroborate prior research emphasizing the impact of weather conditions and driver behavior on road safety, several contributing factors exhibited variations from previously reported studies. These discrepancies can be attributed to distinct dataset characteristics, geographical considerations, temporal variations, lower speed limits in the study area, and the implementation of specific safety measures, such as the widespread presence of enforcement cameras within London.

For instance, this research analysis uncovered that adverse weather conditions, particularly heavy rainfall and fog, showed a heightened association with accident rates, surpassing the influence reported in broader-scale studies. Moreover, driver behavior, although consistently identified as a contributing factor in road safety, displayed nuanced trends within London's A-Roads.

These variations underscore the imperative nature of tailoring road safety strategies to local conditions and conducting granular analyses specific to regional contexts. The predictive models, structured around these localized dynamics, offer a robust methodology applicable to corridor or area-based analyses. The insights gleaned from this study hold immense potential in informing nuanced policy decisions and formulating targeted road safety measures aimed at curbing accidents on London's busiest roads. Furthermore, these insights may have broader applicability, extending beyond A-Roads to diverse road types and regions with similar contextual characteristics.

However, it's crucial to acknowledge the limitations inherent in this study. Primarily focusing on A-Roads in London might limit the generalizability of our findings to other road classifications or cities worldwide. Additionally, the reliance on police-reported data, while comprehensive, might overlook unreported or minor traffic incidents, potentially influencing the accuracy of our analyses.

Moving forward, future research endeavors could explore expanding the scope beyond A-Roads to encompass a wider spectrum of road types and geographical regions. Exploring alternative data sources, such as crowd-sourced or sensor-generated data, could address the limitations of police-reported data, providing a more comprehensive understanding of traffic accidents and their underlying causes.

In conclusion, the study's findings underscore the significance of tailoring road safety strategies to local conditions and offer robust predictive models that hold promise for informing policy decisions. By acknowledging regional nuances and leveraging predictive analytics, our research contributes valuable insights that transcend the confines of London's A-Roads, potentially informing road safety initiatives worldwide.

## 7 Recommendations

1. **Broadening the Scope:** While this study focused on A-roads, the methodology and insights can be applied to other road types. Researchers and policymakers can use similar approaches to understand and predict accidents on B-roads, highways, and city streets.
2. **Global Applicability:** The principles and methods used in this study can be adapted for cities worldwide. However, it's crucial to consider features such as local factors, cultural differences, and driving behaviours when applying the model to different regions.
3. **Prioritizing Measures:** For vulnerable such as pedestrians, cyclists, and motorcyclists:
• Enhance road infrastructure, including better pedestrian crossings, dedicated cycle lanes and facilities, and improved way finding/signage.
• Launch awareness campaigns to educate drivers about the importance of sharing the road and being vigilant.
• Implement stricter regulations, monitoring and penalty enforcements, especially in areas with high active modes presence.

## Data availability statement

Sharing research data helps other researchers evaluate your findings, build on your work and to increase trust in your article. We encourage all our authors to make as much of their data publicly available as reasonably possible. Please note that your response to the following questions regarding the public data availability and the reasons for potentially not making data available will be available alongside your article upon publication.

## CRediT authorship contribution statement

**Mohammad Balawi:** Writing – review & editing, Writing – original draft, Visualization, Validation, Software, Methodology, Investigation, Formal analysis, Data curation, Conceptualization. **Goktug Tenekeci:** Validation, Supervision, Project administration, Formal analysis, Conceptualization.

## Declaration of competing interest

The authors declare that they have no known competing financial interests or personal relationships that could have appeared to influence the work reported in this paper.
